# Influence of ear tags on the results of body composition analysis in mice

**DOI:** 10.1002/ame2.12450

**Published:** 2024-06-14

**Authors:** He Liu, Yinghua Zhang, Peng Zhang, Weiping Teng, Zhongyan Shan, Yushu Li, Dan Wang

**Affiliations:** ^1^ Department of Endocrinology and Metabolism and the Institute of Endocrinology, NHC Key Laboratory of Diagnosis and Treatment of Thyroid Diseases The First Hospital of China Medical University Shenyang China; ^2^ School of Public Health China Medical University, China Medical University Shenyang China; ^3^ Shenyang Food and Drug Inspection Institute Shenyang China

**Keywords:** body composition analysis, ear tagging, labeling method, stainless steel, toe clipping

## Abstract

**Background:**

The aim of this study was to investigate the influence of marking methods on the outcomes of body composition analysis and provide guidance for the selection of marking methods in mouse body composition analysis.

**Methods:**

Male C57BL/6J mice aged 6 weeks were randomly assigned for pre‐ and post‐ ear tagging measurements. The body composition of the mice was measured using a small animal body composition analyzer, which provided measurements of the mass of fat, lean, and free fluid. Then, the mass of fat, lean and free fluid to body weight ratio was gained. Further data analysis was conducted to obtain the range and coefficient of variation in body composition measurements for each mouse. The distribution of fat and lean tissue in the mice was also analyzed by comparing the fat‐to‐lean ratio.

**Results:**

(1) The mass of all body composition components in the ear tagging group was significantly lower than that in the control group. (2) There was a significant increase in the range and coefficient of variation of body composition measurements between the ear tagging group and the control group. (3) The fat‐to‐lean ratio in the ear tagging group was significantly lower than that in the control group.

**Conclusions:**

Ear tagging significantly lowered the results of body composition analysis in mice and higher the results of measurement error. Therefore, ear tagging should be avoided as much as possible when conducting body composition analysis experiments in mice.

## Graphical abstract

We investigated the impact of ear tagging on the results of mouse body composition analysis and found that ear tagging significantly decreased the accuracy of the measurements. Therefore, we recommend avoiding the use of ear‐tagged mice for body composition analysis measurements.

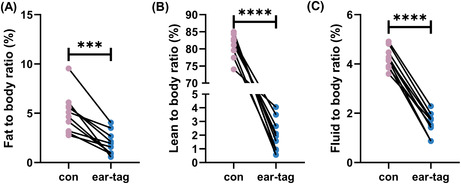

## 1 INTRODUCTION

In recent years, there has been increasing policy attention on the prevention and treatment of obesity. The “Healthy China 2030” plan emphasizes to “strengthen the prevention and treatment of common diseases such as myopia and obesity among students.” The World Health Organization has pointed out that the number of obese people worldwide has nearly tripled since 1975 and has developed the “Global Action Plan on Physical Activity 2018–2030: More Active People for a Healthier World.” It is evident that the prevention, treatment, and research of obesity have received widespread attention both domestically and internationally.

Research on obesity often requires long‐term observation and monitoring. For example, it takes about 2 weeks to 12 months to study the effects of exercise training on overweight and obese individuals.^1^ In animal studies of obesity models, longitudinal observation is often necessary, such as monitoring the body composition and metabolic activity of the animals.^2^

Currently, two main types of in vivo body composition analyzers are commonly used in animal research. One type is the dual‐energy X‐ray absorptiometry (DEXA) that measures the difference between high‐energy and low‐energy X‐ray images. The other type is the small‐animal nuclear magnetic resonance (NMR) that measures the signal differences in each tissue using a magnetic field.^3^ Compared to DEXA, NMR is nondestructive, fast, and highly sensitive. Time‐domain NMR (TD‐NMR) is an NMR technique based on relaxation time detection. This method has advantages such as short measurement time, high repeatability, low cost, noninvasiveness, and high accuracy and sensitivity compared to traditional NMR.^4^

The C57BL/6J mouse strain is prone to developing obesity, metabolic syndrome, fatty liver, and diabetes, making it an excellent choice for developing disease models. It is also a suitable model for studying human monogenic obesity, as a stable adult obesity model can be achieved by feeding them a high‐fat diet for 10 weeks.^5^ In long‐term model construction, clear and stable marking of the mice is particularly important. Common methods for marking mice include dyeing, toe clipping, ear notching, and ear tagging. Because C57BL/6J mice have black fur, dyeing is usually not used for marking. Toe clipping allows for marking within the range of 0–99 and should be performed within the first week after birth. Ear notching allows for marking within the range of 0–400, but the small size of the mouse ears makes it difficult to distinguish the marks, and they can easily be damaged by cage mates, making it unsuitable for long‐term modeling. Ear tagging allows for marking within the range of 1–99 999 and is convenient to use, but the tags cannot be reused, making it expensive. For developing obesity models in C57BL/6J mice, toe clipping and ear tagging are the more suitable methods. However, when studying obesity‐related factors such as muscle strength, toe clipping as a marking method may potentially affect the collection of these.^2^ Ear tags are typically made of 304 stainless steel and are labeled using laser technology. 304 stainless steel is comprised of 70% iron, 20% chromium, and 10% nickel, and metals containing nickel are generally considered to be nonmagnetic or weakly magnetic.^6^ Research has shown that 304 stainless steel can cause minor imaging defects in magnetic resonance imaging (MRI).^7^ Whether the use of stainless‐steel ear tags in mice affects the results of body composition analysis is still unknown. This study aims to compare the results of body composition analysis in C57BL/6J mice before and after ear tagging to investigate whether ear tagging have an impact on the results.

## 2 MATERIALS AND METHODS

### 2.1 Animals and study design

A total of 10 male C57BL/6J mice aged 5 weeks at arrival were purchased from Beijing HFK Bioscience Co., Ltd. (Beijing, China; product number: 11001A) and were fed for 1 week. These mice were used in the experiment at 6 weeks of age, with an average weight of ~18.95 g. All mice were provided with free access to food and water and were maintained under a 12‐h light–dark cycle at a laboratory temperature of 22–25°C and a humidity of 60%. The mice were housed at the Shenyang Institute for Food and Drug Control (Shenyang, China; certificate number: SYXK Liao 2020–0003). The food was provided by Liaoning Changsheng Biotechnology Co., Ltd. (Shenyang, China; certificate number: SYXK Liao 2020–0002). All animal experiments were approved by the Institutional Animal Care and Use Committee at China Medical University and conformed to the existing current animal welfare guidelines (Shenyang, China; certificate number: IACUC TZ2020110).

### 2.2 Materials and instruments

Mouse ear tag forceps and mouse ear tags were bought from Globalebio Technology Co., Ltd. (Beijing, China; GERM‐01, GERM‐02). Body Composition Analysis of Mice and Rats was purchased from Bruker Minisp LF50 (Bremen, Germany).

### 2.3 Body composition analysis of mice

At 6 weeks of age, the body composition of the mice was assessed. Initially, the animal body composition analyzer was calibrated and zeroed. Ten mice were then randomly measured. Subsequently, the mice were placed in a container, and the container was adjusted to ensure complete immobilization of the mice. Initially, their body composition was directly measured without prior marking. Each mouse underwent three measurements, and the results of the body composition analysis (including fat mass, lean mass, and free fluid mass) were documented. After the initial measurement, the mice were promptly ear tagged, and their body composition was reassessed. Each mouse underwent six measurements, with no more than a 10‐min interval between the first and last measurements for each mouse.

### 2.4 Further analysis of the results of body composition analysis

The values obtained from three measurements of the same body composition were averaged to obtain the average values for fat mass, lean mass, and free fluid mass of mice. The average of fat mass, lean mass, and free fluid mass of mice was divided by body weight to obtain the proportion of fat, lean, and free fluid. The range between the values obtained from the three measurements of the same body composition was calculated to obtain the measurement differences for fat mass, lean mass, and free fluid mass. The coefficient of variation between the values obtained from the three measurements of the same body composition was calculated to obtain the degree of measurement dispersion for fat mass, lean mass, and free fluid mass. The calculated ratios of fat mass to lean mass were used to determine the fat‐to‐lean ratio in mice.

### 2.5 Statistical analysis

All values are presented as individual values. The two ends of the line represent the same mouse before and after ear tagging. Statistical analysis was performed using GraphPad Prism 8, and all data were analyzed using paired independent samples *t*‐test. *p* < 0.05 was considered statistically significant.

## 3 RESULTS

### 3.1 The impact of ear tags on mouse body composition analysis

**FIGURE 1:**
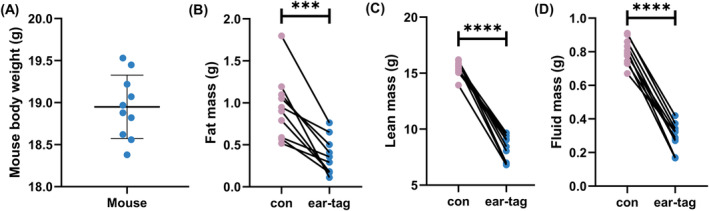
Body composition mass of mice. (A) Mouse body weight. (B) Fat mass. (C) Lean mass. (D) Fluid mass. ***p < 0.001 and ****p < 0.0001.

**FIGURE 2:**
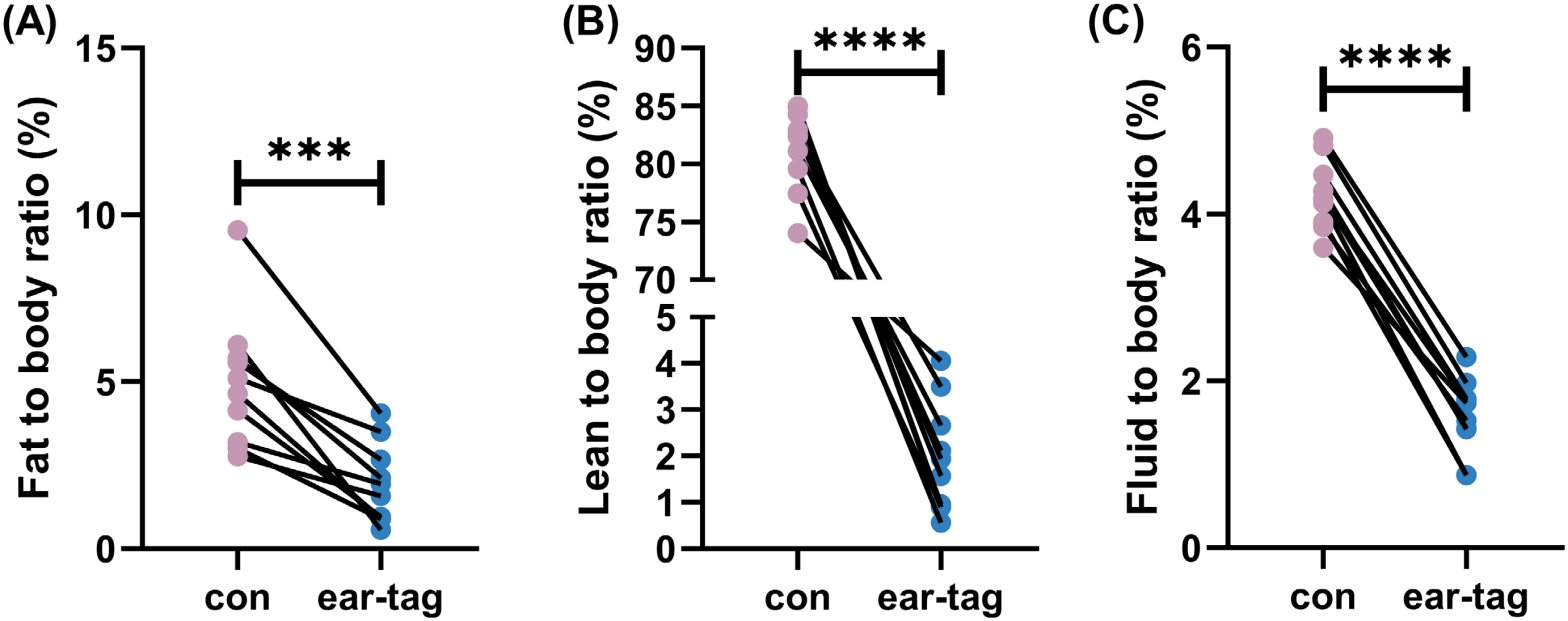
Body composition‐to‐body ratio of mice. (A) Fat to body ratio. (B) Lean to body ratio. (C) Fluid to body ratio. ***p < 0.001 and ****p < 0.0001.

Ear tags had a significant impact on the results of body composition analysis in mice. The weights of mice were shown in Figure 1A. Compared to the control group, the ear‐tag group showed a significantly lower fat mass (*p <* 0.001; Figure 1B), lean mass (*p <* 0.0001; Figure 1C), and free fluid mass (*p <* 0.0001; Figure 1D). Furthermore, the ear‐tag group showed a significantly lower fat‐to‐body ratio (*p <* 0.001; Figure 2A), lean‐to‐body ratio (*p <* 0.0001; Figure 2B), and free fluid‐to‐body ratio (*p <* 0.0001; Figure 2C) as opposed to the control group.

### 3.2 The impact of ear tags on the discrepancy in mouse body composition measurements

**FIGURE 3:**
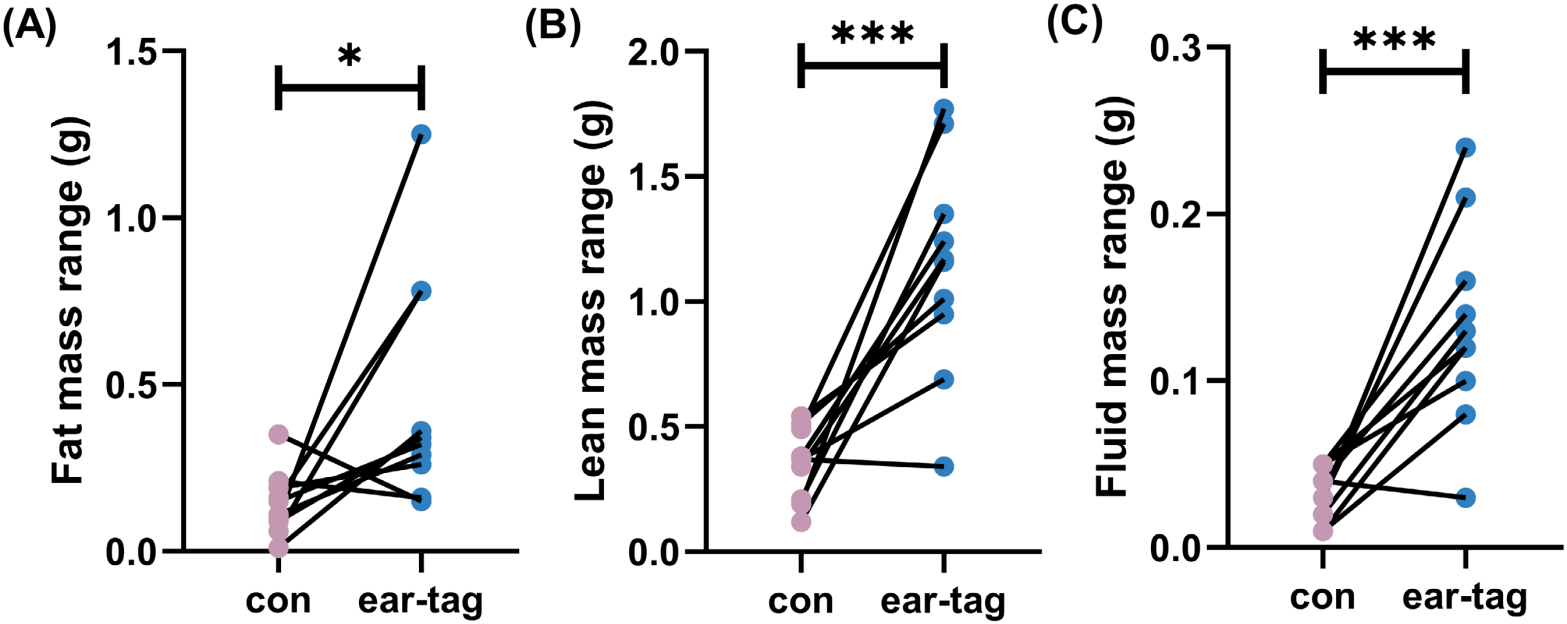
Body composition mass range of mice. (A) Fat mass range. (B) Lean mass range. (C) Fluid mass range. *p < 0.05 and ***p < 0.001.

**FIGURE 4:**
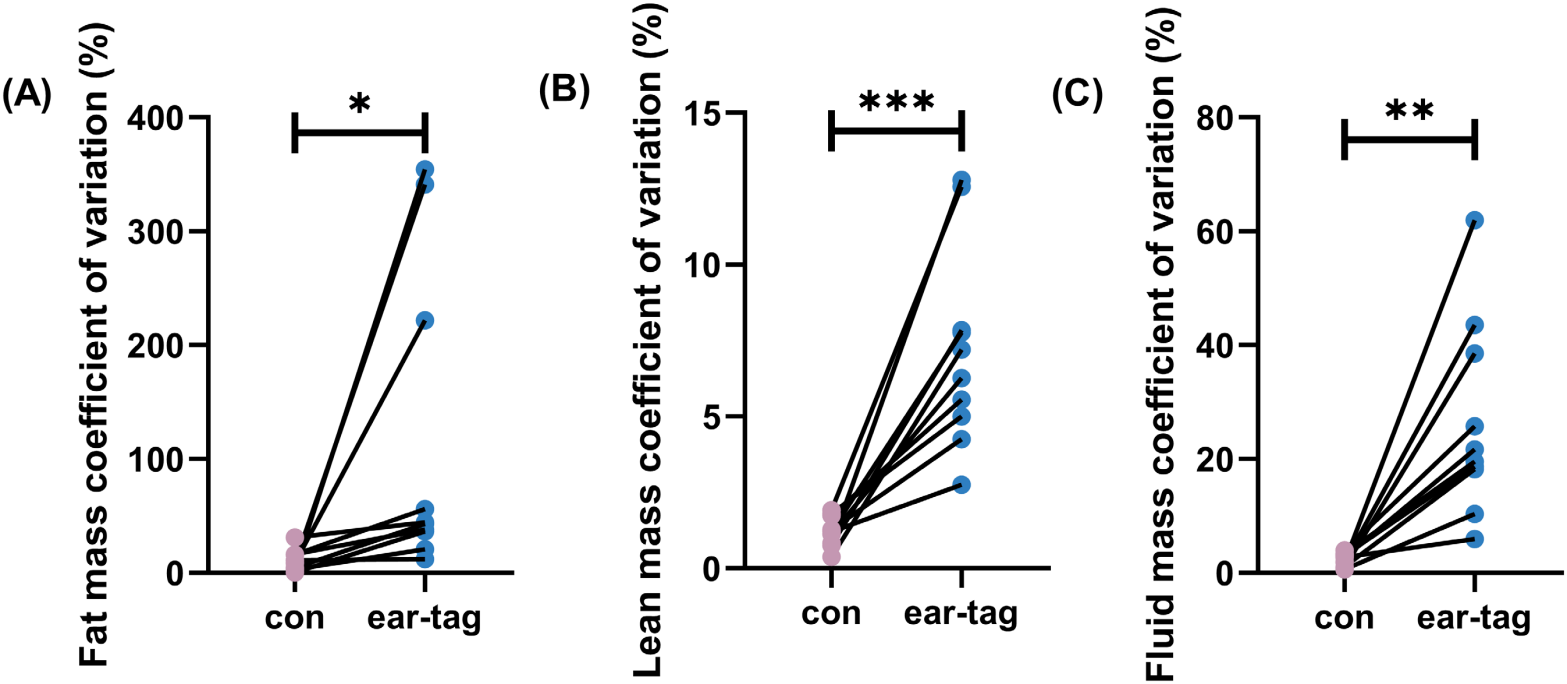
Body composition mass coefficient of variation in mice. (A) Fat mass coefficient of variation. (B) Lean mass coefficient of variation. (C) Fluid mass coefficient of variation. *p < 0.05 and **p < 0.01 and ***p < 0.001.

Ear tags had a significant impact on the results of the measurement range of body composition in mice. Compared to the control group, the ear‐tag group showed a significantly higher measurement range of fat mass (*p <* 0.05; Figure 3A), lean mass (*p <* 0.001; Figure 3B), and free fluid mass (*p <* 0.001; Figure 3C). Furthermore, the ear‐tag group showed a significantly higher measurement coefficient of variation in fat mass (*p <* 0.05; Figure 4A), lean mass (*p <* 0.001; Figure 4B), and free fluid mass (*p <* 0.01; Figure 4C) as opposed to the control group.

### 3.3 The impact of ear tags on the fat‐to‐lean ratio in mice

**FIGURE 5:**
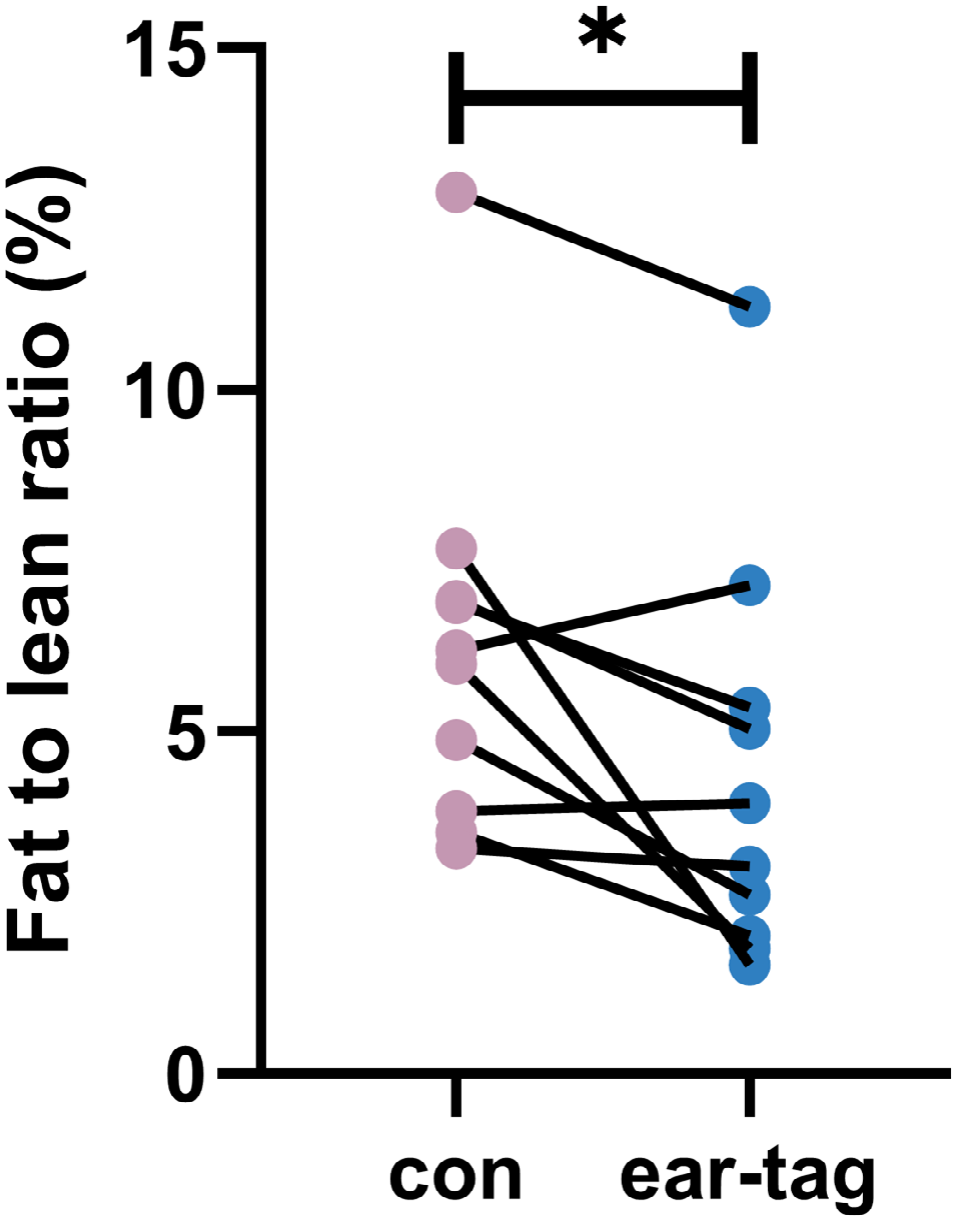
Fat‐to‐lean ratio of mice. *p < 0.05.

Ear tags also had a significant impact on the fat‐to‐lean mass ratio in mice. Compared to the control group, the ear‐tag group showed a significant decrease in the fat‐to‐lean mass ratio (*p* < 0.05; Figure 5).

### 3.4 Comparison of magnetic resonance intensity images before and after ear tagging

**FIGURE 6:**
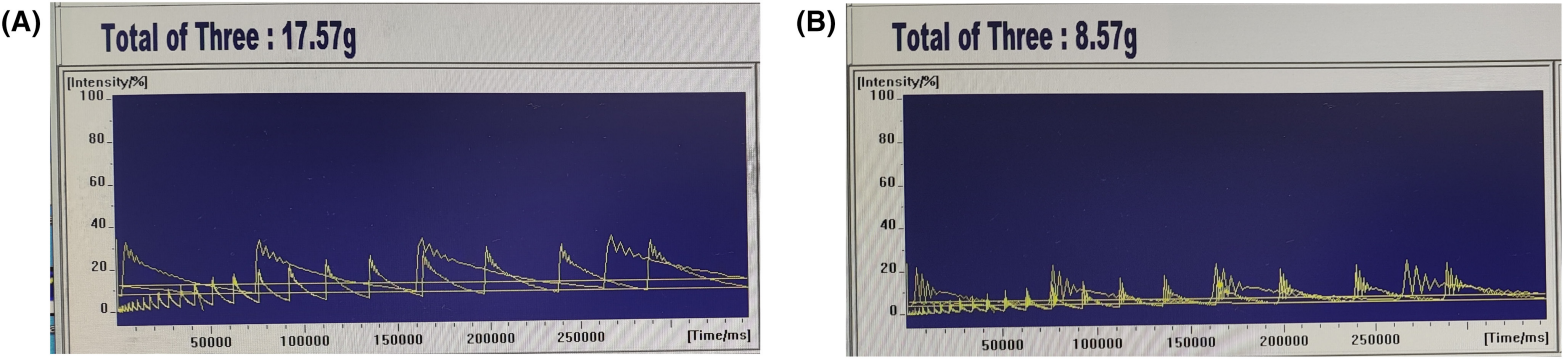
Magnetic resonance intensity images. (A) Magnetic resonance intensity image before ear tagging. (B) Magnetic resonance intensity images after ear tagging.

MRI shows that the ear‐tagged group (Figure 6B) has a smaller area under the curve compared to the control group (Figure 6A), with a shorter period, faster frequency, and a lower maximum signal intensity.

## 4 DISCUSSION

This experiment used stainless‐steel ear tags to label C57BL/6J male mice and applied the body composition analyzer to measure the differences in body composition before and after ear tagging. It was found that the mice labeled with stainless‐steel ear tags had significantly lower body composition and body composition ratio after ear tagging compared to before. These results indicated that ear tags can interfere with the measurement of mouse body composition and cause a consistent decrease in the results. Additionally, stainless‐steel ear tags caused a significant increase in the variation in measurement errors. The experimental results demonstrate that ear tags can consistently affect the decrease in body composition analysis results and the increase in the measurement errors.

MRI uses magnetic fields to emit radio waves and collects data using a computer to reconstruct images of human organs and tissues. The images obtained are further analyzed by the computer to obtain detailed data. The advantages of MRI are its nonionizing and noninvasive nature and high resolution, and its applications are becoming increasingly widespread.^8^ TD‐NMR is a technique based on the principles of low‐field magnetic resonance, which requires only a permanent magnet to perform relevant measurements based on relaxation time. TD‐NMR is inexpensive and convenient compared to traditional NMR. TD‐NMR technology detects bacteria in food rapidly, sensitively, and specifically.^9^ The advantages of TD‐NMR have increased its use in metabolism‐related research. Studies have used TD‐NMR technology to explore hepatic steatosis in high‐fat‐fed rats and mice, and it can detect an increase in liver lipid concentration in rats fed a high‐fat diet for 12 weeks, as well as liver changes in genetically obese mice.^10^

Stainless steel, a type of metal alloy, is typically nonmagnetic or weakly magnetic.^7^ Stainless‐steel sutures are used for reconstructing cartilaginous structures in the ear. Whether patients with such sutures can safely undergo MRI remains unknown. Previous studies have investigated the effects of stainless‐steel sutures on MRI and found that at a magnetic field strength of 3T no significant displacements or temperature increases were observed in the stainless‐steel sutures. Additionally, the artifacts were relatively small and limited to the vicinity of the stainless‐steel sutures.^11^

Research has also found differences in the safety of intrauterine devices (IUD) during MRI measurements. IUDs primarily composed of copper or gold are safe for MRI and exhibit minimal imaging artifacts. However, stainless‐steel IUDs demonstrate significant displacement forces, torque, and noticeable artifacts, indicating that stainless‐steel IUDs are not safe for MRI.^12^ Wang et al. discovered that the artifacts caused by stainless‐steel orthodontic appliances in MRI imaging can be corrected using a permanent magnet, effectively improving image inhomogeneity and geometric distortion.^13^ Breit et al. investigated whether the artifacts from different metal joint implants varied with different magnetic field strengths and metals. They found that the artifacts from stainless steel were significantly smaller at magnetic field strengths of 0.55 and 1.5T compared to those of 3T.^14^

Most metals cause artifacts only in the vicinity of the MRI imaging. For example, in a case where stainless‐steel orthodontic brackets were accidentally swallowed, resulting in artifacts in the rectum during MRI, the cause of an unknown rectal artifact discovered during an examination for unexplained abdominal pain was later determined to be due to the ingestion of stainless‐steel orthodontic brackets. This demonstrates that MRI artifacts are often related to the location of the metal.^15^

MRI can accurately and directly measure muscle and fat, enabling faster and more convenient measurement of body composition.^16^ We found that most studies on animal body composition analysis do not mention the labeling method for mice. For example, the study conducted by Hadjihambi et al. mentioned the detection of mouse body composition only at 0 and 16 weeks, without specifying the specific labeling method.^17^

The body composition analyzer reads the masses of three different components based on the different relaxation times of different tissues under the low‐field magnetic field produced by permanent magnets, although the magnetic field in the low field is relatively less affected by metals. The size of mouse ear tags is generally small, about one‐fourth of a mouse's ear, and the components of ear tags are mostly alloys. Considering the material and size of mouse ear tags themselves, it is inferred that their impact on the results of mouse body composition analysis should be relatively small. However, this study found that using stainless‐steel ear tags would significantly lower the measurement results. This indicates that when conducting long‐term longitudinal experiments to observe mice, it is necessary to avoid using stainless‐steel ear tags for marking.

## 5 CONCLUSIONS

In summary, this experiment analyzed the impact of stainless‐steel ear tags on mouse body composition analysis results, indicating that ear tags are not suitable for body composition analysis experiments. This lays the theoretical basis for subsequent related experiments.

## AUTHOR CONTRIBUTIONS

He Liu conceived and designed this study; He Liu and Peng Zhang were in charge of animal experiments; He Liu, Peng Zhang, Yinghua Zhang, and Dan Wang were involved in animal breeding and experiments. He Liu drafted the manuscript; He Liu and Yushu Li conducted data analysis; Yushu Li, Weiping Teng, and Zhongyan Shan proofread the manuscript. All authors read and approved the manuscript.

## FUNDING INFORMATION

None.

## CONFLICT OF INTEREST STATEMENT

The authors declare that there are no conflicts of interests regarding the publication of this paper.

## ETHICS STATEMENT

All mice were purchased from Beijing HFK Bioscience Co., Ltd (Product number: 11001A). All mice were provided with free access to food and water, were maintained under a 12h light/dark cycle with a laboratory temperature of 22–25°C and humidity of 60%. The mice were housed at the Shenyang Institute for Food and Drug Control (Liaoning, China, certificate number: SYXK Liao 2020‐0003). The food was provided by Liaoning Changsheng Biotechnology Co., Ltd (Liaoning, China, certificate number: SYXK Liao2020‐0002). All animal experiments were approved by the Institutional Animal Care and Use Committee at China Medical University and conformed to the existing current animal welfare guidelines (Liaoning, China, certificate number: IACUC TZ2020110).
